# Occupational self-efficacy, job satisfaction and learning potential of the workplace in a sample of diabetes specialist nurses: A structural equation modeling analysis

**DOI:** 10.1016/j.ijnsa.2025.100446

**Published:** 2025-11-02

**Authors:** Alma Dautovic, Ulla Fredriksson-Larsson, Kajsa Yang Hansen, Eva Brink

**Affiliations:** a University West, Department of Health Sciences, Gustava Melins Gata 2, Trollhättan S-461 32, Sweden; b University of Gothenburg, Department of Education and Special Education, Box 100, Gothenburg S-405 30, Sweden

**Keywords:** Diabetes mellitus, Health Personnel, Mediation analysis, Self-efficacy, Path analysis, Work-integrated learning

## Abstract

**Background:**

Diabetes mellitus is a chronic condition, cases of which are expected to continue rising worldwide. Diabetes specialist nurses play an essential role by assisting patients with preventing or delaying disease complications. Research has suggested an association between occupational self-efficacy and job satisfaction among professionals. However, the relationship between these factors among diabetes specialist nurses, as well as the learning potential of the workplace in this context, remains unexplored.

**Objective:**

This study aimed to explore the relationships between occupational self-efficacy, the learning potential of the workplace and job satisfaction.

**Design:**

A cross-sectional study design was used.

**Setting:**

The study data were collected through a national online survey conducted in Sweden.

**Participants:**

A total of 157 registered nurses who provide diabetes care to patients were included.

**Methods:**

Data were obtained through an online survey with a response rate of 28%. All variables were measured using Swedish-translated standardised instruments. The model was constructed and tested using structural equation modeling analysis with the hypothesis that perceived occupational self-efficacy has both direct and indirect effects on diabetes specialist nurses' job satisfaction and is mediated by the learning potential of the workplace.

**Results:**

The findings supported the hypothesised model. The total effect of the relationship between occupational self-efficacy and job satisfaction was 0.547 (*p* < .001), comprising both the direct effect (β = 0.359, *p* < .0001) between these constructs and the indirect effect (0.188, *p* < .001). The indirect pathways included occupational self-efficacy, which was statistically significantly associated with all three dimensions of the *Learning Potential of the Workplace* scale: *Opportunity to reflect* (β = 0.480, *p* < .0001), *Support in learning* (β = 0.226, *p* < .01), and *Time for exploration* (β = 0.330, *p* < .0001). Both *Support in learning* and *Time for exploration* were also statistically significantly associated with job satisfaction (β = 0.236 and β = 0.266, respectively, *p* < .01), thereby contributing to the sum of the indirect effect (0.188, *p* < .001). One dimension, *Time for exploration*, was identified as a mediator between occupational self-efficacy and job satisfaction, which explained 9% (*p* < .01) of the variance in job satisfaction.

**Conclusions:**

These results demonstrated the association between occupational self-efficacy and job satisfaction among diabetes specialist nurses. The mediation effect of the *Time for exploration* dimension underscores the importance of providing sufficient time for workplace learning. This finding suggests that fostering a supportive learning environment could be associated with job satisfaction.

**Registration:**

Not registered.

**What is already known**

• Research has shown a relationship between self-efficacy and job satisfaction in various professions other than diabetes specialist nurses.
• Diabetes specialist nurses play a vital role in preventive diabetes care, yet they find their role challenging. This challenge necessitates that they continually acquire competence and knowledge to fulfil their professional responsibilities.

Alt-text: Unlabelled box

**What this paper adds**

• Occupational self-efficacy was associated with job satisfaction among diabetes specialist nurses.
• *Time for exploration*, as a dimension of the *Learning Potential of the Workplace* scale, mediated the relationship between occupational self-efficacy and job satisfaction, thus implying the importance of management providing sufficient time for workplace learning.

Alt-text: Unlabelled box

## 1 Introduction

Diabetes mellitus is a chronic condition, cases of which have increased in recent decades; moreover, the incidence rate is expected to continue to rise. Persons with diabetes face the risk of various severe and life-threatening complications, which can result in a heightened demand for medical attention, a decline in quality of life and an increased risk of early death. However, if appropriate diabetes management is achieved, these severe complications can be delayed or prevented altogether (International Diabetes Foundation, 2021).

Evidence has demonstrated that interventions led by diabetes specialist nurses are vital in helping patients with diabetes to persistently lower their blood pressure, blood sugar levels (glycated haemoglobin), LDL cholesterol, and triglycerides as well as to improve their overall control of diabetes and its associated risk factors (Gadot and Azuri, 2023). Diabetes specialist nurses in Sweden have described their professional role as multifaceted and involving a range of different responsibilities, such as having expertise in diabetes care and acting as a teacher. The nurses have also highlighted the importance of acquired knowledge and skills in diabetes care. However, they perceive it as challenging to prioritise their learning due to their perception that management assigns a low priority to ongoing nurse education. As a result, diabetes specialist nurses in Sweden feel they are at risk of not always feeling sufficiently informed (Boström et al., 2012).

In a related study, diabetes specialist nurses reported having higher learning and quantitative demands than other healthcare professionals, with these high learning demands correlating to their professional role conflict. One possible explanation is that diabetes specialist nurses in Sweden often work independently, with limited interaction or support from colleagues apart from consulting physicians (Boström et al., 2013). This conflict underscores the need to recognise hospitals and clinics as learning environments for healthcare professionals to facilitate work-integrated learning.

Work-integrated learning encompasses diverse forms of learning that are integral to professional work practice (Björck and Willermark, 2024). The workplace can be an ideal space for professional development, because it merges learning with work while providing disciplinary knowledge and the social intelligence for successful collaboration. As workplaces are considered dynamic learning environments, it is essential to understand how the learning potential of the workplace—a multidimensional concept that encompasses interactional learning from colleagues and supervisors as well as task-related learning through reflection and experimentation (Nikolova et al., 2014)—both enhances and contributes to continual professional learning. Employees who proactively gain relevant knowledge and skills at work become valuable assets for the organisation they serve. However, rapid organisational developments have made it challenging for employees to acquire vocationally relevant knowledge, skills and attributes through the exercise of personal initiative in their workplaces. In addition, despite the increasing interest in workplace learning, the mechanisms that support this learning remain unexplored (Nikolova et al., 2014).

In particular, to promote professional growth and job satisfaction among nurses, research has suggested that organisations’ should create a work environment that ensures a balanced workload and reserves sufficient time for ongoing professional development (Zhao et al., 2025). Job satisfaction refers to how much individuals enjoy their work. Locke (1976) defined job satisfaction and job dissatisfaction as emotional states that result from the perceived relationship between one’s expectations of one’s job and the appraisal of different aspects of the job. A quantitative systematic review and meta-analysis identified educational interventions that improved nurses’ job satisfaction and suggested that organisations should focus on strategies that foster employees’ self-motivation (Niskala et al., 2020). In addition, research has shown that job satisfaction and self-efficacy are positively associated (Bargsted et al., 2019).

Self-efficacy is defined as an individuals’ perceptions of their ability to fulfil a task under the prevailing conditions (Bandura, 1997). Occupational self-efficacy refers to the competence that individuals feel concerning their ability to successfully accomplish the tasks involved in their job (Rigotti et al., 2008). Workplace leadership plays a crucial role in enhancing employees’ occupational self-efficacy, which not only improves their confidence in performing job-related tasks but also positively influences their readiness for and engagement with occupational transitions (Schyns, 2004). Yunis et al. (2024) demonstrated that a practice education program tailored for professionals improved their self-efficacy and perceived quality of care, leading to a positive impact on metabolic markers in patients with type 2 diabetes.

A systematic review of qualitative studies showed that diabetes care has advanced over the past few decades and led to changes in the roles of professionals in this field. As a result, professionals, such as diabetes specialist nurses, need ongoing professional development to update their knowledge. Indeed, inadequate skills are identified as the main barrier to managing diabetes care (Sibounheuang et al., 2020). Additionally, another study found that only a small proportion of diabetes specialist nurses reported having a dedicated budget or protected time for ongoing professional development. The nurses highlighted limited support from managers and the absence of dedicated learning time as key barriers to their professional growth (Riordan et al., 2017). At the same time, Shiri et al. (2023) showed in their systematic review that healthcare workers who receive ongoing development and training opportunities will experience higher job satisfaction and provide improved patient care. In addition, a major reason for turnover among nurses is a lack of development opportunities.

Previous research has demonstrated associations between occupational self-efficacy and job satisfaction in various professions and work contexts; however, available insights specifically regarding diabetes specialist nurses are limited. Therefore, this study aimed to explore the relationships between occupational self-efficacy, the learning potential of the workplace and job satisfaction among diabetes specialist nurses. The following hypotheses were proposed.

H1 Occupational self-efficacy is positively associated with job satisfaction.

H2 The three dimensions of the *Learning Potential of the Workplace* (*Opportunity to reflect, Support in learning*, and *Time for exploration*) mediate the association between occupational self-efficacy and job satisfaction.

## 2 Methods

### 2.1 Study design

This study employed a cross-sectional quantitative design and followed the Strengthening the Reporting of Observational Studies in Epidemiology reporting checklist for cross-sectional studies (von Elm et al., 2007).

### 2.2 Sampling and participants

The participants were recruited nationally through the Swedish Diabetes Nurses’ Interest Association membership database. The association facilitated all contact with potential participants, distributed the online survey and provided study information to a total of 560 potential participants on behalf of the research team. To qualify for inclusion in the study, participants were required to engage with persons with diabetes in their daily work. Based on the sample-size calculation for a structural equation model, the actual sample size of 157 participants had sufficient statistical power (Soper, 2025).

### 2.3 Measures

#### 2.3.1 Demographic information

The participants completed a brief demographic survey that was used to collect gender, age, educational background, years of professional experience in diabetes care and current workplace setting.

#### 2.3.2 Occupational self-efficacy

Schyns and von Collani (2002) introduced the *Occupational Self-Efficacy Scale*, which was eventually condensed into a shorter version of eight items. Rigotti and colleagues (2008) further validated an even more succinct version comprising six items in five countries, including Sweden. The participants in this study rated their responses on a six-point Likert scale ranging from (1) ‘not at all true’ to (6) ‘completely true’. The higher the total score, the higher the participant’s perception of individual occupational self-efficacy. For this dataset, Cronbach’s α coefficient was determined to be 0.88, indicating good reliability.

#### 2.3.3 Learning potential of the workplace

The instrument known as *Learning Potential of the Workplace* consists of 12 items. This original version consisted of four dimensions: *Learning through reflection, Learning through experimentation, Learning from colleagues*, and *Learning from superviso*r (Nikolova et al., 2014). The original instrument was translated and validated in a Swedish sample of diabetes specialist nurses, which resulted in a changed factor structure with three dimensions labelled *Opportunity to reflect, Support in learning*, and *Time for exploration*. The participants rated their responses using a five-point Likert scale that ranged from (1) ‘not applicable’ to (5) ‘completely applicable’ (Dautovic et al., 2025). A high total score reflected a high perceived learning potential of the workplace. For this dataset, Cronbach’s α coefficients for the dimensions were as follows: *Opportunity to reflect*, 0.87; *Support in learning*, 0.84; and *Time for exploration*, 0.98.

#### 2.3.4 Short index of job satisfaction

The original version of the *Index of Job Satisfaction* comprised 18 items (Brayfield and Rothe, 1951). A shorter version, the *Short Index of Job Satisfaction,* used in the present study, was condensed and evaluated to measure job satisfaction through one dimension including five items (Sinval and Marôco, 2020). This instrument was translated into Swedish (using the forward-backward translation process) and validated in this study’s sample of diabetes specialist nurses. The participants rated each item using a five-point Likert scale ranging from (1) *‘*strongly disagree’ to (5) ‘strongly agree’. A high total score reflected high reported perceived job satisfaction. Two items (item 3, ‘each day at work seems like it will never end’ and item 5, ‘I consider my job to be rather unpleasant’) were reversed before analysis. The Cronbach’s α coefficient for this dataset was 0.80.

### 2.4 Data collection

**Fig. 1:**
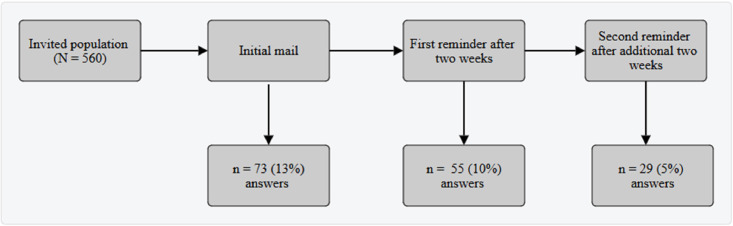
Flowchart of the sampling process leading to a total of 157 study participants.

Data were collected nationally via an online questionnaire distributed to diabetes specialist nurses by the Swedish Diabetes Nurses’ Interest Association between May and July 2023, using their email list. Following the initial invitation, two reminder emails were sent by the association that resulted in 157 completed responses—a response rate of 28 % (Fig. 1). The questionnaire was hosted on a secure digital platform and took participants an average of 22 min to complete.

To ensure that each participant submitted only one response, email verification was required. This measure effectively prevented duplicate entries; no duplicate or missing data were recorded. Email addresses were treated as personal data, coded with unique identifiers, and stored separately from the survey responses to maintain confidentiality.

### 2.5 Statistical analysis

The analyses were conducted within the structural equation modeling framework, which allows for the evaluation of theoretical models by investigating the relationship between the theoretical constructs of interest (i.e. latent variables or factors) and their indicators, and among a set of theoretical constructs (Hu and Bentler, 1999). These analyses were carried out using Mplus version 8.1 statistical analysis software (Muthén and Muthén, 1998–2017).

Following Anderson and Gerbing (1988), a two-step analytic approach was employed. The construct’s validity was first examined using confirmatory factor analysis for the constructs *Occupational Self-Efficacy,* the three dimensions of *Learning Potential of the Workplace* and the *Short Index of Job Satisfaction*. In the second step, a path model was estimated to examine the interrelationships between these latent constructs, where the outcome variable is regressed on the independent variables directly or indirectly through the mediators (Baron and Kenny, 1986).

To establish a mediation effect in a path analysis, three criteria need to be met: a) the independent variable must significantly account for variability in the dependent variable, b) the independent variable must significantly account for variability in the mediator, and c) the mediator must significantly account for variability in the dependent variable when controlling for the independent variable. In addition, the effect of the independent variables on the dependent variable should decrease when the mediator enters simultaneously (Baron and Kenny, 1986; Preacher and Hayes, 2008).

To evaluate whether the proposed theoretical model properly represented the underlying structure of the data, a set of global goodness-of-fit indices was used, including the chi-square (χ²) goodness-of-fit statistic along with the degree of freedom (df) of the model, the root mean square error of approximation (RMSEA), the comparative fit index (CFI), and the standardised root mean squared residuals (SRMR) (Hu and Bentler, 1999). The cutoff values that indicated a good model fit of the hypothesised model were *p* ≤ 0.01 for χ² or χ²/df ≤ 5, RMSEA ≤ 0.08, CFI ≥ 0.95, and SRMR ≤ 0.08 (Hu and Bentler, 1999).

A significance level of *p* ≤ 0.05 was set to determine the statistical significance of the relationships between the study variables, e.g. the path coefficients between the dependent variable job satisfaction, the independent variable occupational self-efficacy, and the mediators of workplace learning potential dimensions (e.g. *Opportunity to reflect, Support in learning*, and *Time for exploration*). The relationship could also be between the theoretical latent factors and their indicators in terms of factor loadings. A factor loading should be higher than 0.30 to be considered adequate. Otherwise, the latent variable indicator should be removed from the measurement model because the latent construct does not explain a meaningful amount of variation in the indicator, even when a factor loading is significant (i.e. *p* ≤ 0.05) (Hooper et al., 2008; Brown, 2015).

### 2.6 Ethical considerations

The Swedish Ethical Review Authority approved the study (Dnr 2023–00,920–01), which adhered to the Declaration of Helsinki for Medical Research Ethics and Standards (World Medical Association, 2024). All participants received detailed letters informing them about the project, their voluntary participation, their right to withdraw at any time, and the researchers' contact details. The participants were informed that completing the online survey signified informed consent for voluntary participation.

## 3 Results

### 3.1 Sample demographic characteristics

The 157 participating diabetes specialist nurses were mainly females (97 %) with a mean age of 51 (*SD* = 9.4) years. The participants were primarily employed in primary healthcare (51.6 %) and hospital care (42.7 %), with a minority in other locations (5.7 %). The participants reported a mean of 12.66 (*SD* = 9.4) years of diabetes-care work experience and estimated their weekly working time as 26.6 (*SD* = 13.1) hours per week in diabetes care. Most had a master’s degree (77.7 %) (Table 1).

**Table 1:** Characteristics of participants.

	*n*	%
**Gender**		
Female	153	97.5
Male	4	2.5
**Age**		
Below 29	3	1.9
30 – 39	19	12.1
40 – 49	43	27.4
50 – 59	56	35.7
60 – 69	36	22.9
**Education**		
Master's degree (one year)	122	77.7
Bachelor's degree	35	22.3
**Workplace**		
Primary Health Care	81	51.6
Hospital Health Care	67	42.7
Municipal Health Care	6	3.8
Digital diabetes care	3	1.9
**Work experience in diabetes care**		
1 – 9 years	69	43.9
10 – 19 years	49	31.2
20 – 29 years	28	17.8
30 – 39 years	8	5.1
40 – 49 years	3	1.9

Note. *N* = 157.

### 3.2 Descriptive statistics and correlations between the constructs

A total summed score was derived for the *Occupational Self-Efficacy* and *Short Index of Job Satisfaction* scales*,* and the three dimensions of the *Learning Potential of the Workplace* instrument. Table 2 presents the correlations among the study variables, along with their means (*M*), standard deviations (*SD*), ranges and confidence intervals (*CI*). Pearson’s correlation coefficients among the summed scores of these latent constructs were statistically significant (*p* < .01). The highest correlation was observed between the *Opportunity to reflect* and job satisfaction (0.577), followed by the correlation between the *Opportunity to reflect* and *Time for exploration* (0.574). The correlation between occupational self-efficacy, on the one hand, and *Support in learning* and *Time for exploration*, on the other hand, was among the lowest, being 0.219 and 0.272, respectively. A multiple regression model was estimated to predict job satisfaction by the rest of the summed scores. No multicollinearity issue was identified as the variance inflation factors ranged from 1.22 to 1.97 among the predictors.

**Table 2:** Descriptive statistics and correlations between study variables.

Variable	M	SD	Theoretical Range	95 % CI	1	2	3	4	5
*1. Occupational Self-Efficacy* *	30.38	3.56	6–36	29.82–30.94	(0.88)				
*2. Opportunity to reflect* *	14.03	3.15	4–20	13.53 – 14.52	.425⁎⁎	(0.87)			
*3. Support in learning* *	20.99	4.71	6–30	20.25 – 21.73	.219⁎⁎	.538⁎⁎	(0.84)		
*4. Time for exploration* *	5.59	1.96	2–10	5.28 – 5.90	.272⁎⁎	.574⁎⁎	435⁎⁎	(0.98)	
*5. Short Index of Job Satisfaction* *	21.34	3.35	5–25	20.81 – 21.87	.454⁎⁎	.577⁎⁎	.440⁎⁎	.538⁎⁎	(0.80)

*Notes: M* = Mean; SD = Standard Deviation; CI = Confidence Interval.

⁎ Summed score of all its indicators.

⁎⁎ Pearson correlation: statistically significant at the 0.01 level (2-tailed). Cronbach’s alpha is in brackets. *N* = 157.

### 3.3 The measurement properties of the constructs

Initially, three measurement models were tested respectively for *Occupational Self-Efficacy, Learning Potential of the Workplace,* and *Short Index of Job Satisfaction*. However, these measurement models did not fit the data well, so model modification was accomplished to improve the model fit. For the *Short Index of Job Satisfaction* model, the factor loadings were below the threshold (< 0.30) for items SIJS3 (‘Each day at work seems like it will never end’) and SIJS5 (‘I consider my job to be rather unpleasant’). This may have been due to these indicators having too many measurement errors and failing to capture enough variation in job satisfaction. Therefore, they were removed from the confirmatory factor analysis model of the *Short Index of Job Satisfaction*.

For the *Learning Potential of the Workplace* model, the three dimensions can be identified, where *Support in learning, Time for exploration* and *Opportunity to reflect* were correlated in an oblique model. According to the modification indices, the model fit was improved by adding a correlated residual between the *Support in learning* items SIL8 and SIL9. The one-factor measurement model of *Occupational Self-Efficacy* reached an acceptable model fit when residual covariance between indicators OSE2 and OSE3 was introduced (Table 3).

**Table 3:** The indicators of each latent construct and the factor loadings.

Construct	Indicators	Factor loadings
*Occupational Self-Efficacy (OSE)*	OSE 1	.699
	OSE 2	.674
	OSE 3	.764
	OSE 4	.633
	OSE 5	.760
	OSE 6	.853
*Opportunity to reflect (OTR)*	OTR1	.764
	OTR2	.886
	OTR3	.848
	OTR4	.700
*Time for exploration (TFE)*	TFE5	.916
	TFE6	.953
*Support in learning (SIL)*	SIL7	.460
	SIL8	.584
	SIL9	.555
	SIL10	.872
	SIL11	.816
	SIL12	.694
*Short Index of Job Satisfaction (SIJS)*	SIJS1	.791
	SIJS2	.926
	SIJS4	.760

**Fig. 2:**
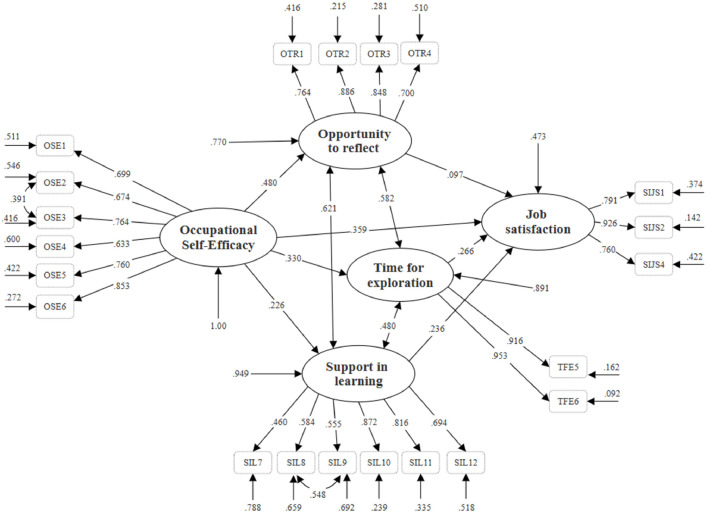
The final model with standardised path coefficients and the direct and indirect effects.

*Note*: Model fit: X^2^=284.68, df=177, CFI = 0.95, RMSEA=0.06, SRMR=0.07, *p* < .0001

Table 4 presents the model fit indices of all three measurement models and indicates that no significant deviation was observed between the model-implied variance-covariance matrix and that observed from the data. While the factor loadings were generally around 0.70 or above for all of these latent constructs, relatively lower estimates were also observed, particularly within the *Support in learning* dimension. However, all of the factor loadings remained well above the criteria of 0.30 (see Fig. 2), thus ensuring the adequacy of the construct validity.

**Table 4:** Model fit indices of the measurement models.

Model fit	X^2^	df	*p*	CFI	RMSEA	SRMR
*Occupational Self-Efficacy*	6.825	8	0.0000	1.000	0.000	0.017
*Learning Potential of the Workplace*	100.594	50	0.0000	0.955	0.080	0.059
*Short Index of Job Satisfaction*	12.808	4	0.0000	0.982	0.118	0.031

### 3.4 The interrelationships between the constructs

A path model was specified to examine the interrelationships between these latent constructs, where *Occupational Self-Efficacy* affected the *Short Index of Job Satisfaction* both directly and indirectly through the three dimensions of the *Learning Potential of the Workplace*. The model explained 54.7 % of the variance in job satisfaction, and the fit indices showed good model fit (χ^2^ = 284.68, df = 177, CFI = 0.95, RMSEA = 0.06 and SRMR = 0.07, *p <* .0001). All of the direct and indirect relationships in the final model are illustrated in Fig. 2 and Table 5*.*

**Table 5:** The final model with standardised path coefficients.

Pathways	Path coefficient		*p*
Direct			
*Occupational Self-Efficacy* → *Short Index of Job Satisfaction*	.359		< .0001
Indirect			
*Occupational Self-Efficacy* → *Opportunity to reflect*→ *Short Index of Job Satisfaction*	.053		NS
*Occupational Self-Efficacy* → *Time for exploration*→ *Short Index of Job Satisfaction*	.088		< 0.01
*Occupational Self-Efficacy* → *Support in learning* → *Short Index of Job Satisfaction*	.047		NS

*Note*. NS= statistically not significant.

A statistically significant path coefficient was observed for the direct relationship between *Occupational Self-Efficacy* and the *Short Index of Job Satisfaction* (β = 0.359, *p* < .0001). Further, *Occupational Self-Efficacy* demonstrated statistically significant associations with all three dimensions of the *Learning Potential of the Workplace* scale: *Opportunity to reflect* (β = 0.480, *p* < .0001), *Support in learning* (β = 0.226, *p* < .01), and *Time for exploration* (β = 0.330, *p* < .0001). *Support in learning* and *Time for exploration* were also statistically significantly associated with the *Short Index of Job Satisfaction*, with the estimated path coefficients being 0.236 and 0.266, respectively (see Fig. 2). Among the mediation effects between *Occupational Self-Efficacy* and the *Short Index of Job Satisfaction* through the three dimensions of the *Learning Potential of the Workplace*, only the indirect effect through *Time for exploration* was statistically significant (β = 0.088, *p <* .05). The total indirect effect was observed to be 0.188 (*p* < .001). The total effect of *Occupational Self-Efficacy* on the *Short Index of Job Satisfaction* was 0.547 (*p* < .001), which corresponded to the sum of the direct and indirect pathways (Table 5).

The findings supported the first hypothesis (H1), which posited the presence of a direct relationship between occupational self-efficacy and job satisfaction. The second hypothesis (H2) was partially supported; that is, one of three dimensions of the *Learning Potential of the Workplace* scale mediated the relationship between occupational self-efficacy and job satisfaction. More explicitly, *Time for exploration* was identified as a mediator between occupational self-efficacy and job satisfaction, with an *R*² of 9 % (*p* < .01).

## 4 Discussion

The results of this study showed that occupational self-efficacy was associated with job satisfaction among diabetes specialist nurses. In addition, we observed that the *Learning Potential of the Workplace* scale dimension, *Time for exploration*, mediated the relationship between occupational self-efficacy and job satisfaction, which suggests that interventions targeting greater time for exploration could potentially increase job satisfaction.

An important finding was the importance of time as crucial for workplace learning in diabetes healthcare. This dimension of the *Learning Potential of the Workplace* scale assesses whether professionals are provided sufficient time to discover how to independently perform tasks more efficiently and explore new solutions for task-related problems on their own. Ensuring that diabetes specialist nurses have adequate time to engage in workplace learning activities is vital for fostering stimulating environments that enhance learning and improve diabetes healthcare practices.

Providing time for learning also allows workplaces to benefit from motivated employees who enhance workflows by independently identifying improvement needs and self-directing their learning according to their discoveries. The motivation for adult learners to engage in workplace learning activities is driven by their need to understand the meaning or significance of these activities to fully engage in them (Illeris, 2003). For work-integrated learning to take place through situated learning when an opportunity for learning arises in workplaces, resources for knowledge acquisition are needed (Pennbrant and Svensson, 2018), which makes it essential to have time to seize the learning opportunities that emerge during the professional workday.

Loeng (2020) stated in a review article that a core concept of adult education is learners’ ability to self-direct their learning such that they take the initiative and define what is worth learning. Learning at the workplace can thus be interpreted as learning for the workplace: the actions initiated by employees serve to improve the skills necessary to perform work tasks (Segers et al., 2022). Workplaces that prioritise learning, exploration and innovation are fundamental for employee engagement, learning and organisational development. Moreover, workplace culture can facilitate or obstruct learning and either engage or disengage employees in workplace learning processes (Ahsan, 2025).

Access to knowledge is strongly influenced by workplace culture, and, to facilitate workplace learning, time and monetary resources are necessary (Pylväs et al., 2022). The findings relative to *Time for exploration* indicated that diabetes specialist nurses are inclined to engage in workplace learning when they discover potential for improvement or solutions in their work tasks. Thus, time must be available for them to pursue this learning when such situations arise spontaneously.

Workplaces are increasingly important places for self-directed continuous learning. The minimum requirement for learning to take place is for learners to have control over the time, pace and place of learning (Loeng, 2020). Lack of time due to job pressures and responsibilities is a well-known inhibitor of workplace learning, which is why a professional-growth strategy and learning-committed leadership are essential. Thus, managers and leaders who create learning opportunities are crucial components (Ellinger, 2005). Workplaces that promote workplace-based learning strategies by allocating meaningful learning time (e.g. *Time for exploration*) may be a successful way to encourage employees’ engagement in workplace learning.

Our findings also indicated that workplace learning potential was associated with the diabetes specialist nurses’ perceived job satisfaction. The results of our study revealed a statistically significant association between occupational self-efficacy and job satisfaction among the nurses who participated. This finding was consistent with previous research that demonstrated similar results. For instance, Bargsted et al. (2019) showed a positive correlation between professional self-efficacy and job satisfaction among participants across various roles and organisations, with a majority employed in healthcare roles.

Other studies have highlighted similar trends among nurses across different age groups working in hospitals. For example, De Simone et al. (2018) reported a positive relationship between self-efficacy and job satisfaction among nurses (mean age = 43.5 years) in inpatient hospital wards. Likewise, Kurniawan et al. (2019) observed a comparable pattern among younger nurses (mean age = 25.1 years) in the same work environment.

However, there is a gap in the literature regarding the relationship between occupational self-efficacy and job satisfaction, specifically within the specialist nurse population. Our findings contribute to this area of research. The structural equation model indicated that occupational self-efficacy explained 35.9 % of the variance in job satisfaction among diabetes specialist nurses. This suggests that a higher level of occupational self-efficacy is likely to predict greater job satisfaction within this professional group.

This insight is crucial to pursue because previous studies showed that higher job satisfaction among healthcare personnel positively impacted the quality of care. Healthcare units where the staff members were satisfied with their jobs reportedly scored high in terms of the quality of care they provided both in hospitals (Chang et al., 2009; Kılıç Barmanpek et al., 2022) and in primary healthcare settings (Szecsenyi et al., 2011). Moreover, nurses’ self-reported job satisfaction has also been shown to predict patient satisfaction (Perry et al., 2018). These insights underscore the importance of fostering occupational self-efficacy among diabetes specialist nurses because it could enhance their job satisfaction and potentially positively influence the diabetes patients’ satisfaction.

Moreover, research on work-integrated learning should emphasise conditions that enable professionals to engage actively in workplace learning throughout their careers. Providing opportunities in the workplace for professionals to continue their learning and education is crucial to ensuring the retention of skilled professionals. Several studies included in a systematic review have shown that insufficient workplace learning contributes to nurses leaving the field (Shiri et al., 2023), which may, in turn, hinder the advancement of diabetes healthcare.

### 4.1 Limitations

The strength of this study lies in its focus on diabetes specialist nurses, who play a vital role in diabetes healthcare. One limitation may be the recruitment of participants through an interest association. This recruitment channel could have affected the representativeness of the study population because the association may attract individuals with particular characteristics to become members. Additionally, while the structural equation modeling analysis was suitable for achieving our study aim, the cross-sectional design showed relationships but precluded causality between variables.

## 5 Conclusion

This study demonstrated an association between occupational self-efficacy and job satisfaction among diabetes specialist nurses. The mediation effect of the *Time for exploration* dimension of the *Learning Potential of the Workplace* scale underscores the importance of providing sufficient time for workplace learning. This finding suggests that fostering a supportive learning environment could be associated with job satisfaction. Given the positive impact of job satisfaction on the quality of care, it is crucial to prioritise strategies that promote diabetes specialist nurses’ occupational self-efficacy. Future research should continue exploring these relationships to further improve healthcare outcomes.

## Ethics statement

The Swedish Ethical Review Authority approved the study (Dnr 2023–00920–01).

## Funding

This research received no specific grant from any funding agency in the public, commercial, or not-for-profit sectors.

## Data availability

Due to ethical restrictions, the participants in this study did not agree to share their data publicly. Therefore, supporting data is unavailable.

## CRediT authorship contribution statement

**Alma Dautovic:** Writing – review & editing, Writing – original draft, Validation, Software, Project administration, Methodology, Formal analysis, Data curation, Conceptualization. **Ulla Fredriksson-Larsson:** Writing – review & editing, Methodology, Formal analysis, Conceptualization. **Kajsa Yang Hansen:** Writing – review & editing, Validation, Formal analysis. **Eva Brink:** Writing – review & editing, Supervision, Methodology, Formal analysis, Conceptualization.

## Declaration of competing interest

The authors declare that they have no known competing financial interests or personal relationships that could appear to have influenced the work reported in this paper.
